# Biomaterials and nanomedicine for bone regeneration: Progress and future prospects

**DOI:** 10.1002/EXP.20210011

**Published:** 2021-10-30

**Authors:** Jun Zhou, Zhongyang Zhang, John Joseph, Xingcai Zhang, Bijan Emiliano Ferdows, Dylan Neal Patel, Wei Chen, Giuseppe Banfi, Roberto Molinaro, Donato Cosco, Na Kong, Nitin Joshi, Omid C. Farokhzad, Claudia Corbo, Wei Tao

**Affiliations:** ^1^ Center for Nanomedicine and Department of Anesthesiology Brigham and Women's Hospital Harvard Medical School Boston Massachusetts USA; ^2^ School of Engineering and Applied Sciences Harvard University Cambridge Massachusetts USA; ^3^ School of Engineering Massachusetts Institute of Technology Cambridge Massachusetts USA; ^4^ Pomona College Claremont California USA; ^5^ Jericho High School Jericho New York USA; ^6^ IRCCS Galeazzi Milano Italy; ^7^ Università Vita e Salute San Raffaele Milano Italy; ^8^ IRCCS San Raffaele Hospital Milano Italy; ^9^ Department of Health Science Campus Universitario‐Germaneto “Magna Græcia” University of Catanzaro Catanzaro Italy; ^10^ School of Medicine and Surgery Nanomedicine Center Nanomib University of Milano‐Bicocca Vedano al Lambro Italy

**Keywords:** biomaterials, bone defects, nanomedicine, scaffolds, tissue engineering

## Abstract

Bone defects pose a heavy burden on patients, orthopedic surgeons, and public health resources. Various pathological conditions cause bone defects including trauma, tumors, inflammation, osteoporosis, and so forth. Auto‐ and allograft transplantation have been developed as the most commonly used clinic treatment methods, among which autologous bone grafts are the golden standard. Yet the repair of bone defects, especially large‐volume defects in the geriatric population or those complicated with systemic disease, is still a challenge for regenerative medicine from the clinical perspective. The fast development of biomaterials and nanomedicine favors the emergence and promotion of efficient bone regeneration therapies. In this review, we briefly summarize the progress of novel biomaterial and nanomedical approaches to bone regeneration and then discuss the current challenges that still hinder their clinical applications in treating bone defects.

## Graphical abstract

We present an overview of common causes of bone defects, the unmet clinical needs, and the utility of biomaterials and nanotechnology to facilitate bone regeneration. Besides, we propose several key approaches to improve bone repair strategies. This perspective provides a guidance for future research direction of bone repair to improve treatment efficiency in both clinical and research perspectives.

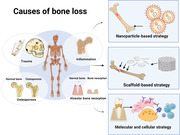

## 1 INTRODUCTION

Skeletal defects are frequently associated with a variety of pathogenic conditions with different causes and clinical outcomes, including trauma (fractures), infections (osteomyelitis and periodontitis), tumors, osteoporosis, and many other bone‐related diseases.^[^ ^1^, ^2^ ^]^ Specifically, bone regeneration in patients with large bone defects presents a vexing and long‐standing puzzle for clinicians and researchers due to the limited regenerative ability of the skeleton. For example, large bone defects resulting from neoplastic resection or surgical excision of infected tissues cannot be spontaneously reconstructed, which leads to poor healing efficiency even with delayed union or non‐union in elderly patients.^[^ ^3^, ^4^ ^]^ This in turn can severely impair patient quality of life or even give rise to life‐threatening situations. Thus, beyond conventional bone grafts, there is an urgent need for advanced and effective therapeutic interventions for large bone defect regeneration.

Recently emerging innovations in the fields of biomaterials and nanomedicine offer great promise in treating bone diseases,^[^ ^5^, ^6^ ^]^ which may actually create a new frontier in bone repair. Integration of biological factors and nanoparticles (NPs) has demonstrated therapeutic applications in bone regeneration and treatment of a wide range of bone diseases.^[^ ^7^, ^8^ ^]^ Moreover, the development of bioscaffolds such as three‐dimensional (3D)‐printed implants avoids the requirement of bone donation, showing great promise as biomaterial‐based tissue engineering (TE) strategy for repairing critical bone defects.^[^ ^9^ ^]^ Herein, we presented an overview of common causes of bone defects, the unmet clinical needs, and the utility of biomaterials and nanotechnology to facilitate bone regeneration. Later, we proposed several key approaches to improve bone repair strategies from both clinical and research perspectives.

## 2 THE CAUSES OF BONE LOSS AND TREATMENT OPTIONS

**FIGURE 1:**
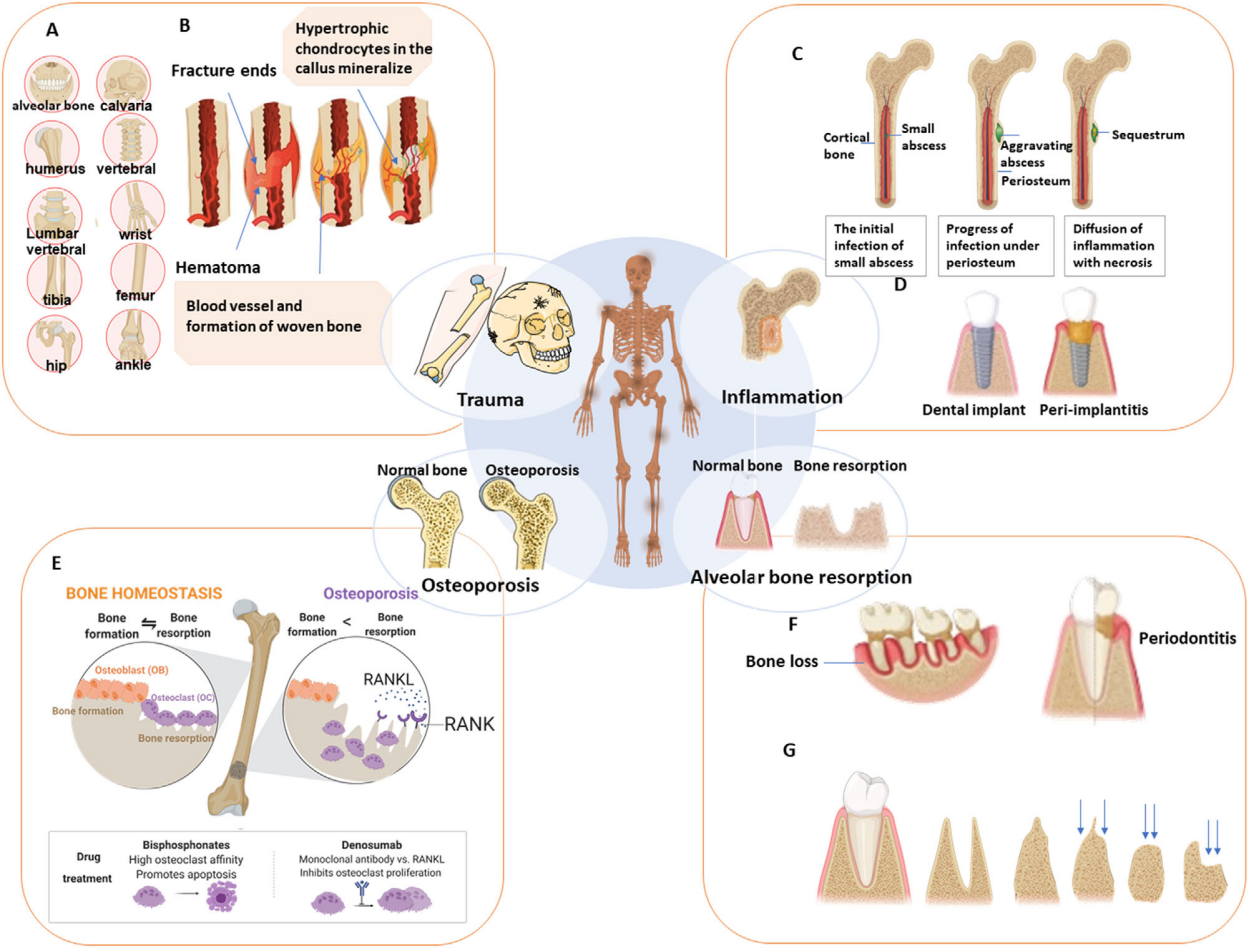
Main causes of bone loss. (A) Common fracture locations include the craniofacial (alveolar bone, calvaria), long bones (femur, tibia, humerus), wrist (radius/ ulna), ankle (above the joint, distal tibia/fibula), and vertebral sites. (B) The process of normal fracture healing through the formation of a cartilaginous callus. (C) Progress of osteomyelitis (OM). A local abscess creates diffuse infection and produces a region of necrotic bone tissue. (D) Unlike non‐inflammatory dental implants (left), peri‐implantitis (right) may lead to loss of supporting tissue and implant failure. (E) Bone homeostasis and common dynamic cycle (left) of bone formation and bone resorption. At right is bone remodeling in osteoporosis, in which bone resorption by osteoclasts exceeds bone formation mediated by osteoblasts. (F) Bone loss as a cause of high‐morbidity periodontitis. (G) Alveolar ridge resorption post‐extraction of the tooth, from left to right, pre‐extraction, post‐extraction, high and well rounded, knife ridge, low and well rounded, and depressed

The clinical outcomes of bone repair are closely tied to the causes and forms of bone damage. Small bone defects can be spontaneously healed without any intervention. In contrast, critical‐sized defects are generally too large to regenerate naturally; examples include limb amputations following bone tumor resection, vehicle accidents with significant bone loss, bone excision after chronic infection, and gunshot wounds.^[^ ^9^, ^10^ ^]^ The abilities of bone to regenerate efficiently, maintain mineralization, and heal after damage depends on its dynamic remodeling capacity. However, the regenerative process is limited by the capacity for self‐renewal, that is, to rebuild bone mass and microstructure. Here we firstly introduce the most frequent challenges for clinical treatment including bone lesions caused by trauma, inflammation, age‐related osteoporosis, and alveolar bone loss in dental medicine, especially aging‐ and tooth‐extraction‐related natural resorption (Figure 1).^[^ ^11^ ^]^ In addition, we present current healing strategies in clinic corresponding to these etiologies.

### 2.1 Trauma

Bone lesions caused by trauma can occur in patients of all ages, which could be a result of traffic accidents, falls of the elderly, or countless other scenarios. Bone fracture is one of the most common modes of trauma and around 8 million people suffer from fractures in the United States alone for each year.^[^ ^12^ ^]^ Bone fracture can distribute among several important anatomical locations across the body,^[^ ^13^ ^]^ as shown in Figure 1A. Among them, fractures at some sites such as long bones (e.g., femur) are usually associated with high morbidity and mortality.^[^ ^14^ ^]^ Besides, up to 10% of the individuals can encounter delayed healing or non‐union,^[^ ^15^ ^]^ particularly in conjunction with pathological conditions such as diabetes,^[^ ^16^ ^]^ age‐related osteoporosis, genetic factors, and infections.

Repairing of bone fracture includes several biological steps as shown in Figure 1B,^[^ ^12^, ^13^ ^]^ in which multiple cellular events (e.g., bone precursors cells, hematopoietic, endothelial, inflammatory cells)^[^ ^17^, ^18^, ^19^ ^]^ and activation of several regulatory genes and bone morphogenetic pathways are involved. There exist diversiform strategies to repair bone damage from trauma, such as synthetic bone grafts and cell‐based therapy, whether in combination with bioactive molecules or alone. However, insufficient clinical efficacy and safety data on these approaches are required to solve the challenges presented by delayed healing and non‐union conditions.^[^ ^13^ ^]^

### 2.2 Inflammation

Inflammatory cytokines, released as a result of activation of several cell types in the immune system, can simultaneously initiate bone degradation besides promoting inflammation.^[^ ^20^ ^]^ Thus, both local and systemic bone loss could be attributed to the body's inflammatory response, which is a non‐negligible cause. Chronic osteomyelitis (OM) refers to infection of the bone marrow which remains difficult to treat with considerable morbidity and high risk of recurrence.^[^ ^21^ ^]^ It commonly implies bacterial infection, typically caused by *Staphylococcus aureus* during an injury or fixation surgery, and even large‐volume bone loss from inflammatory bone resorption. The infection can be expanded to several locations in bone, and eventually produce necrotic bone, termed a sequestrum,^[^ ^22^ ^]^ as shown in Figure 1C. Surgical removal of infected bone is considered as the best means in clinic to clear the focus of infection, followed by the administration of antibiotics to improve the prognosis of OM. Bone grafts or prosthetic implants are commonly used as substitutions for bone loss. However, even with proper treatment, OM has a high recurrence rate and risk of chronicity,^[^ ^23^ ^]^ due to as‐developed antibiotics resistance of certain bacterial species^[^ ^22^ ^]^ and peri‐implantitis (Figure 1D).

Therefore, the specific etiology of inflammation‐derived bone defects places multiple design requirements on nanomedical materials, including the capability of tackling both inhibitions of inflammatory responses and promotion of bone tissue regeneration. For instance, nanocarriers were developed for sustained release of antibiotics in a precisely defined manner in clinical treatment for OM.^[^ ^24^ ^]^ Besides, a better understanding of the molecular mechanisms regulated by bacteria to stimulate infection should be clarified, and concurrently find the way to block the inflammation progress.

### 2.3 Osteoporosis

**FIGURE 2:**
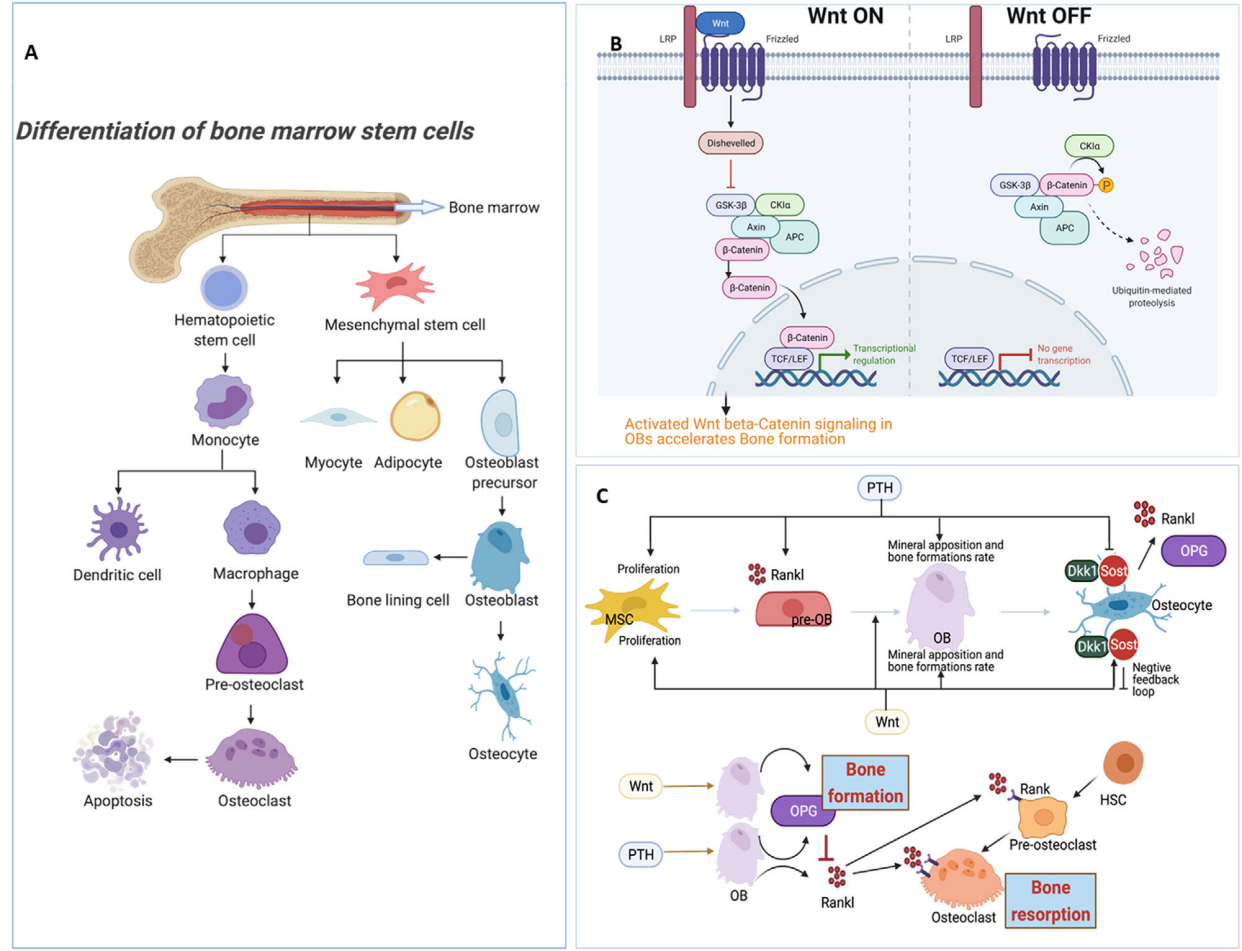
The key mechanisms of cellular and molecular biology in bone. (A) Differentiation process of osteoblasts and osteoclasts. Bone marrow mesenchymal stem cells (MSCs) are multipotent cells with the capacity to differentiate toward osteoblast (OB), chondrocyte, and adipocyte lineages. The largest cellular population in bone, osteocytes are derived from OBs. Osteoporosis represents an increase in bone marrow fat tissue due to a shift in the differentiation of MSCs to adipocytes rather than to osteoblasts. Therefore, the fate of MSCs affects the proportion of various types of bone cells and bone mass overall. On the other hand, osteoclasts (OCs), which facilitate bone resorption, originate from the monocyte‐macrophage lineage. (B) Effects of the Wnt β‐catenin signaling pathway. The Wnt/β‐catenin signaling pathway is transduced by the stabilization of β‐catenin following the interaction between a specific Wnt ligand and its designated receptors. Wnt has been considered the main regulator of osteogenesis. The activation of the Wnt signaling pathway can promote the formation of bone. (C) Cross‐talk and effects of the two key pathways in bone metabolism (PTH and Wnt signaling) on MSC, pre‐OB, OB, and indirect function on osteoclasts. Both PTH and Wnt promote the proliferation of MSCs and the commitment of these cells to the OB lineage, whereas PTH can also stimulate OB to produce RANKL, which facilitates pre‐OC differentiation to OC. The Wnt pathway produces osteoprotegerin (OPG), hindering OC differentiation and function by suppressing RANKL. Sclerostin (Sost) and Dkk1, activated by Wnt, can suppress Wnt activity, creating a negative feedback loop

Osteoporosis is an age‐related systemic skeletal disorder, characterized by low bone mineral density (BMD) and elevated risk of fracture. Osteoporosis is an enormous and growing public health problem, particularly among postmenopausal women. Osteoporosis causes approximately 9 million fracture cases annually in the world,^[^ ^25^ ^]^ while one‐third women over age 50 years experienced osteoporotic fractures.^[^ ^26^ ^]^ The pathogenesis of osteoporosis is predominantly associated with bone homeostasis, the balance of bone remodeling between formation and resorption by specific cells (Figure 1E) including osteoblasts(OBs), osteocytes, and osteoclasts (OCs). Mesenchymal stem cells (MSCs) in the bone marrow are the precursor of osteoblasts, as well as adipocytes, chondrocytes, and myocytes (Figure 2A),^[^ ^27^ ^]^ hence the fate of MSCs are deeply involved in the pathogenesis of osteoporosis.

The Wnt/β‐catenin pathway and calcium signaling through parathyroid hormone (PTH) are the most well‐studied and essential regulatory pathways in bone (Figure 2B,C). The transcription factors activated in these main anabolic pathways regulate the function and differentiation of progenitor cells toward OBs to improve bone formation.^[^ ^28^ ^]^ On the other hand, OCs derived from the macrophage lineage could lead to bone resorption, which is regulated by osteoclast differentiation macrophage colony‐stimulating factor (M‐CSF), receptor activator of nuclear factor‐κB ligand (RANKL), and various cytokines.^[^ ^27^ ^]^ The Wnt pathway can increase bone mass by activating bone formation and repressing bone resorption, whereas PTH will promote osteoclastogenesis, stimulating the differentiation of pre‐OCs to mature OCs, by enhancing RANKL production from OBs (Figure 2C).^[^ ^29^ ^]^ Over the last few decades, on the basis of understanding the pathogenesis and molecular regulatory mechanism, the most effective pharmaceutical treatments for osteoporosis have relied on anti‐resorptive, osteoclast‐targeting therapies, such as bisphosphonates and denosumab (Figure 1E).

### 2.4 Alveolar bone resorption

Bone loss that mainly refers to bone resorption in the maxilla and mandible continues to be an unresolved issue in dental medicine. Alveolar bone resorption usually arises from age‐dependent bone resorption in older patients, periodontitis‐derived bone loss caused by infection (Figure 1F), and facial bone resorption in post‐extraction sites (Figure 1G). Periodontitis and periodontal atrophy are two major lesions contributing to bone loss in dentistry, leading to residual ridge resorption, especially in older populations.^[^ ^30^ ^]^ According to one report on the prevalence of periodontitis, about 50% of US adults have been affected by periodontitis,^[^ ^31^ ^]^ and 10% of them even suffer severe periodontitis.^[^ ^32^ ^]^ Progressive periodontitis aggravates bone resorption and tooth mobility, resulting in abscesses and severe cases, loss of teeth. Since alveolar bone is the supportive and mechanosensitive tissue surrounding the teeth, it is continuously remodeled under the required masticatory force. Yet the lack of periodontal mechanotransduction after infection on the periodontal tissue induces disuse‐atrophy and extra bone resorption.

With increasing age and repeated tooth extraction, resorption of alveolar bone occurs in the horizontal dimension and vertical ridge height on the facial and buccal aspect of the ridge, termed residual ridge resorption (Figure 1G).^[^ ^33^ ^]^ Because of insufficient bone support to stabilize the prosthesis post‐extraction, most complete dentures will become unstable and loosen, inducing sustaining pain or failed fixation of the denture. Besides, given the current popularity of dental implant restoration, surgical bone tissue augmentation around implants has been applied to achieve better clinical outcomes, producing greater market demand for bone regeneration in dentistry. In addition, one noteworthy factor in dental bone regeneration is the difference in the reconstruction rate of long bone and flat bone.^[^ ^34^ ^]^ Therefore, there is tremendous potential for alveolar bone tissue engineering applications that remain largely uninvestigated.

## 3 BIOMATERIALS AND NANOMEDICINE FOR BONE REPAIR

Bone defects with worse outcomes (i.e., delayed or unpredictable bony healing or high rates of infection) remain big challenges for orthopedic trauma surgeons. Among currently developed treatments, autogenous bone graft and allograft are regarded as the essential standard for bone repair in clinic;^[^ ^35^ ^]^ however, their efficiency is still limited by several shortcomings, such as lack of available donor source.^[^ ^36^ ^]^ To promote the curing effectiveness, the rapid development of biomaterials and nanomedicine during last few decades paves new road for renovating bone regeneration methods. Here we summarized commonly used strategies, including nanoparticle carrier, engineered scaffold, and novel molecular and cellular strategies.

### 3.1 Nanoparticle‐based strategies

**FIGURE 3:**
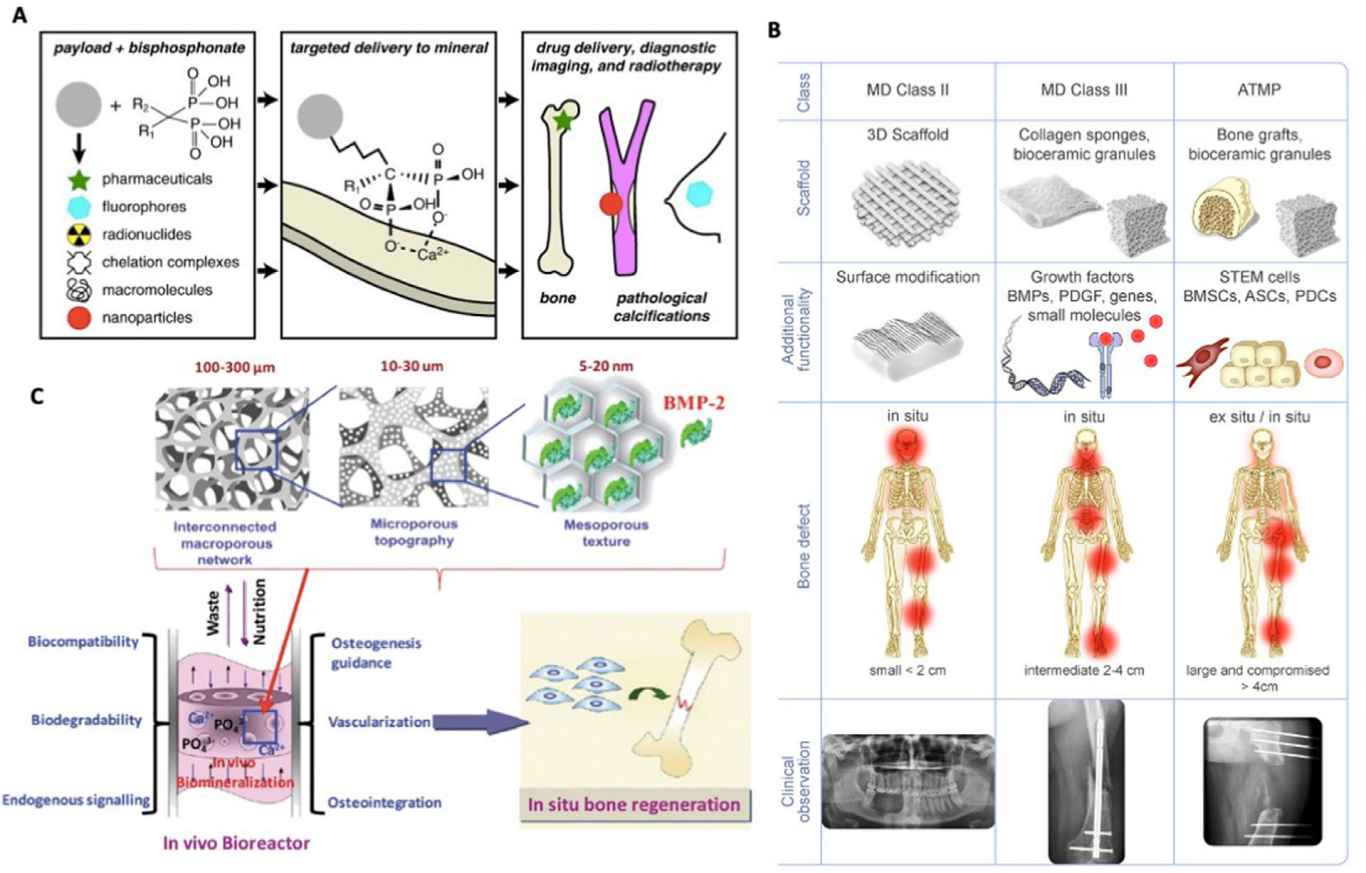
Nanomedicine and biomaterials for bone repair. (A) Utility of bisphosphonates for targeted bone mineral delivery in its unconjugated/ conjugated form to various pharmaceuticals, nanoparticles, fluorophores, chelation complexes, and macromolecules. Bisphosphonates can also be used for drug delivery, radiotherapy, and diagnosis. Reproduced with permission.^[^ ^39^ ^]^ Copyright 2015, Elsevier. (B) The three main scaffold fabrication techniques for bone regeneration. The first uses synthetic bone graft substitutes for medical device(MD) class II, the second a synergy of bioactive molecules in a ceramic scaffold for MD class III, and the third a tissue engineering‐based approach with stem cells in a scaffold, with or without bioactive molecules called advanced therapeutic medicinal products (ATMP). Reproduced with permission.^[^ ^13^ ^]^ Copyright 2018, Elsevier. (C) The development of nanomaterials with biomimicry considering various factors such as interconnected macropores, microporous topography, composition, mineralization, and use of bioactive molecules. These nanomaterials function as in vivo bioreactors for in situ bone regeneration. Reproduced with permission.^[^ ^8^ ^]^ Copyright 2017, RSC Pub

Nanoparticles (NPs) are the central element of nanomedicine, offering unique properties, including a high surface area to volume ratio, along with better chemical and physical performance than their bulk counterparts.^[^ ^37^, ^38^ ^]^ NPs were widely investigated as delivery vehicles or imaging probes for regenerative therapeutics, such as nanoscale ceramic particles, poly(lactic‐*co*‐glycolic) acid (PLGA), gelatin, collagen, and chitosan are excellent materials that are commonly used in bone regeneration. For instance, current strategies for osteoporosis are to develop osteoporotic bone‐targeting drug delivery systems with better therapeutic efficacy but reduced non‐targeted adverse effects. Bisphosphonates, one of the bone‐targeting drugs, can act as a linker agent between NPs and bone minerals due to their high binding affinity with hydroxyapatites.^[^ ^39^ ^]^ (Figure 3A) An alternative strategy for osteoporosis is to modulate cellular and molecular function in bone by delivering osteoclast inhibitors or osteoblast activators using NPs. In the clinic, PLGA, hyaluronic acid, or chitosan NPs have been used for bone‐targeted delivery of small‐molecule drugs, growth factors, or gene‐targeted compounds to promote bone formation. In addition, co‐delivery of multiple drugs (e.g., osteoclast inhibitors and osteoblast activators) in nanocarriers via different functionalizations, can be expected as an all‐in‐one multifunctional strategy for tailoring the balance of bone remodeling.

### 3.2 Scaffold‐based strategies

Autograft and allograft are commonly used strategies in the clinic for large bone defect repair. To address the limitation (source, size, etc.) and function loss of bones in the donor sites, bone graft substitutes (BGSs) such as engineered scaffolds were developed to counteract bone defects. 3D printing of bioinspired materials has been considered as powerful technique to construct customized scaffolds with multifunctional biocompatible compounds (collagen and hydroxyapatite). For instance, novel 3D‐printed poly (lactic acid) (PLA) scaffold functionalized with bioinspired surface coatings further increased the success rate of bone‐implanted devices.^[^ ^40^ ^]^ The surface coatings composed of collagen, minocycline, and citrate‐hydroxyapatite nanoparticles could reduce the formation of bacterial biofilm. The scaffold not only provided 3D structural supporting with adaptable degradation rate, but also released drugs to promote cellular infiltration and mineralization. In addition, 4D bioprinting has been developed to produce dynamic 3D‐patterned biological architectures with stimuli‐responsive materials, which exhibited changeable shapes under various certain stimuli.^[^ ^41^ ^]^

On the other hand, the combination of scaffolds with NPs or biological morphogenetic molecules such as bone morphogenetic protein 2 (BMP‐2) can be a pragmatic approach to achieve multiple therapeutic goals. A variety of scaffold‐based strategies are employed in the restoration of different‐scale damage, as depicted in Figure 3B: (i) synthetic scaffolds, (ii) scaffolds combined with active molecules, and (iii) tissue‐engineered/cell‐seeded scaffolds.^[^ ^13^, ^42^ ^]^ Identifying the appropriate bioactive molecules, their optimal concentrations, and release kinetics from the scaffold presents a clinical challenge related to their high cost, instability, and side effects. Many studies have demonstrated the therapeutic benefits of biological factors within scaffolds in recruiting endogenous host cells. Complete regeneration of critical‐sized bone defects is accomplished by the use of bone structure‐mimicking scaffolds along with the incorporation of proper biomolecules or growth factors. For example, engineered scaffolds with hierarchical architectures and the capability of delivering morphogenetic molecules BMP‐2, or cell‐homing growth factors including VEGF and stromal‐derived growth factor‐1 have been demonstrated promising effects on promoting large bone defect regeneration. These bone‐mimicking construction strategies mainly focus on the understanding of microenvironmental factors such as mineral composition, crystallinity, micro‐porous architecture, and growth factor release. This approach paved the way for the development of a “real” biomimetic novel nanomaterial for in situ bone regeneration (Figure 3C).^[^ ^8^ ^]^

### 3.3 Novel molecular and cellular strategies

Furthermore, superior bone healing with shorter recovery periods can be achieved by utilizing novel molecular and cellular factors. PTH, the most essential regulatory factor affecting bone formation through the calcium‐phosphate metabolism, can activate the Wnt β‐catenin pathway that is vital for the promotion of osteogenesis and inhibition of osteoclastogenesis (Figure 2C). Crosstalk between the PTH and Wnt pathways in different bone cells is key for bone anabolism and might be the best answer for the modulation of bone metabolism. One common production of PTH analogs, PTH (1–34), was initially tested in long‐bone defects in a sheep model and shown to play a pivotal role in osteoconductive and osteoinductive effects.^[^ ^43^ ^]^

A better understanding of the biological factors involved is required to explain these positive outcomes. For instance, getting a clearer picture of the role of inflammatory cytokines and immune cells in bone regeneration will help us understand how to regulate inflammation during bone repair. Moreover, the fate of progenitor cells such as MSCs could direct the differentiation ability of osteogenesis and the outcome of bone regeneration by the influence of materials.^[^ ^44^, ^45^ ^]^ Therefore, future directions should include the regulation of bone remodeling by targeting the molecular and cellular mechanisms of MSCs/osteoblasts/osteoclasts, in order to induce cell‐specific activating or blocking signaling to optimize bone repair. Nanocarriers as a fast‐developing tool in nanomedicine enable on‐target delivery of small molecules to regulate corresponding molecular mechanisms, offering treatment options either by systemic administration or local drug delivery for molecular and cellular strategies.

## 4 PERSPECTIVES

In summary, bone defects arise from various diseases or injuries that proposed different requirements for the development of materials and healing strategies for restoring the bone structure and functions. Additionally, the combination of geriatric population or systemic disease conditions, such as aging and diabetes, greatly impairs bone self‐renewal, with substantial impacts on the therapeutic efficiency. These demands have focused research toward the investigation of novel multifunctional biomaterials, advanced bone/endothelium‐targeting techniques, and combined therapies, aiming for more effective and comprehensive treatments.

### 4.1 Multifunctional materials and combined therapies

Regarding bone diseases, the application of multifunctional materials should combine systemic and local therapies with diverse modes including multi‐drug, co‐delivery, co‐treatment with radiotherapy, ultrasound, and hyperthermia. For bone defects with concomitant inflammation in situ or co‐factors (such as diabetes) that always increase the difficulty of healing, thus, the above aspects of diverse modes should be exploited as much as possible in combination therapies for bone repair.

**FIGURE 4:**
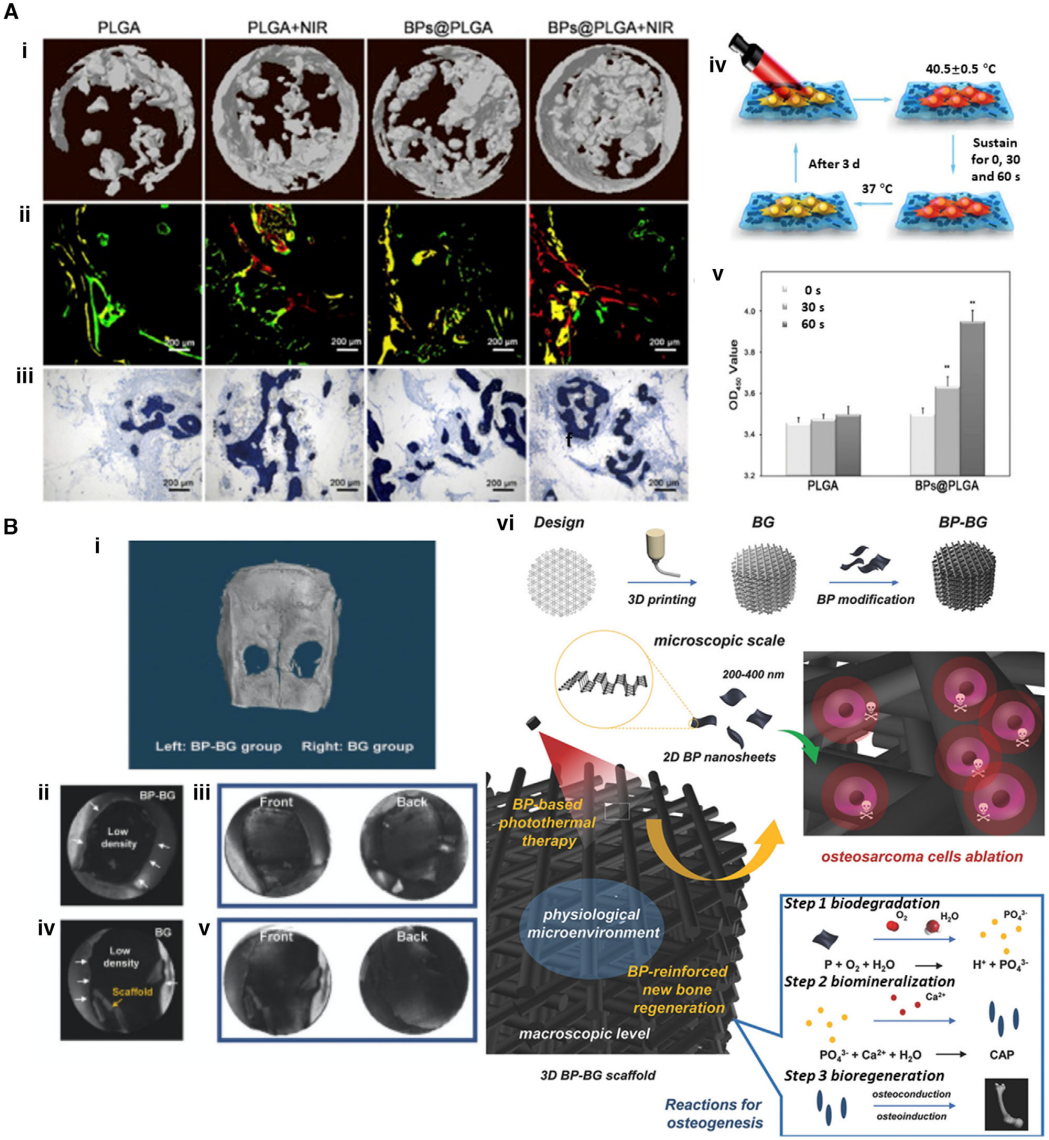
Multifunctional materials and combination therapies by Black phosphorus (BP). (A) Application of BP in bone regeneration using the photothermal conversion of BP under near‐infrared (NIR), with PLGA and BPs@PLGA. (i) 3D reconstruction of bone by Micro‐CT. (ii) Sequential fluorescence labeling of newly formed bone. (iii) Representative histologic graphs with toluidine blue staining. (iv) MSCs cultured on BPs@PLGA membrane with NIR irradiation. (v) Cell viability analysis for biocompatibility of MSCs on PLGA and BPs@PLGA with or without NIR irradiation. ** denotes *p*  <  0.01 compared with the PLGA group. Reproduced with permission.^[^ ^13^ ^]^ Copyright 2019, Elsevier. (B) In vivo osteogenesis performance of the bifunctional 3D‐printed bioglass (BG) scaffold designed using 2D BP nanosheets. (i–v) Micro‐CT 3D imaging from rat calvaria 8 weeks after implantation. Bone defects were restored with BP‐BG scaffold (left) and BG scaffold (right) as control. (i) 3D reconstruction image of micro‐CT in calvaria. The two defects in calvaria represent newborn tissues in the middle of holes that were too thin to be identified by the 3D reconstruction software. BP‐BG group (ii,iii) and BG group (iv,v) were acquired using black (ii,iv) and white (iii,v) substrates. When the color of substrates reverses from black to white, the newborn osseous tissue can be visualized. (vi) Schematic illustration of the fabrication process for BP‐BG scaffold and the stepwise therapeutic strategy for the ablation of osteosarcoma and in situ osteogenesis. Reproduced with permission.^[^ ^65^ ^]^ Copyright 2018, John Wiley and Sons

For instance, immunotherapy has been introduced to enhance therapeutic efficacy in specific cases: functions of macrophages have been investigated in bone tissue engineering for controlling immune response and preventing bone resorption. Two‐dimensional (2D) materials such as photothermal therapeutic agents have found a wide of applications in nanomedicine.^[^ ^46^, ^47^, ^48^, ^49^, ^50^, ^51^, ^52^, ^53^, ^54^, ^55^, ^56^, ^57^ ^]^ Black phosphorus (BP), is an emerging 2D crystalline material with a unique layered structure, holding great promise for bone regeneration due to its excellent biocompatibility, adjustable bandgap, and high photothermal conversion efficiency.^[^ ^58^, ^59^, ^60^, ^61^, ^62^ ^]^ Therefore, BP has been widely applied in the fields of cancer therapy, anti‐inflammation, and bone regeneration using the high‐efficiency photothermal conversion of BP under near‐infrared (NIR) irradiation (Figure 4A).^[^ ^63^, ^64^ ^]^ It has also been reported that a bifunctional 3D‐printed BP‐bioglass (BG) scaffold stimulates anti‐cancer and osteogenesis in situ via the biomineralization of phosphorus‐driven, calcium‐extracted effects under photothermal therapy (Figure 4B).^[^ ^65^ ^]^

### 4.2 Targeting endothelium in the skeleton to alleviate the bone loss

**FIGURE 5:**
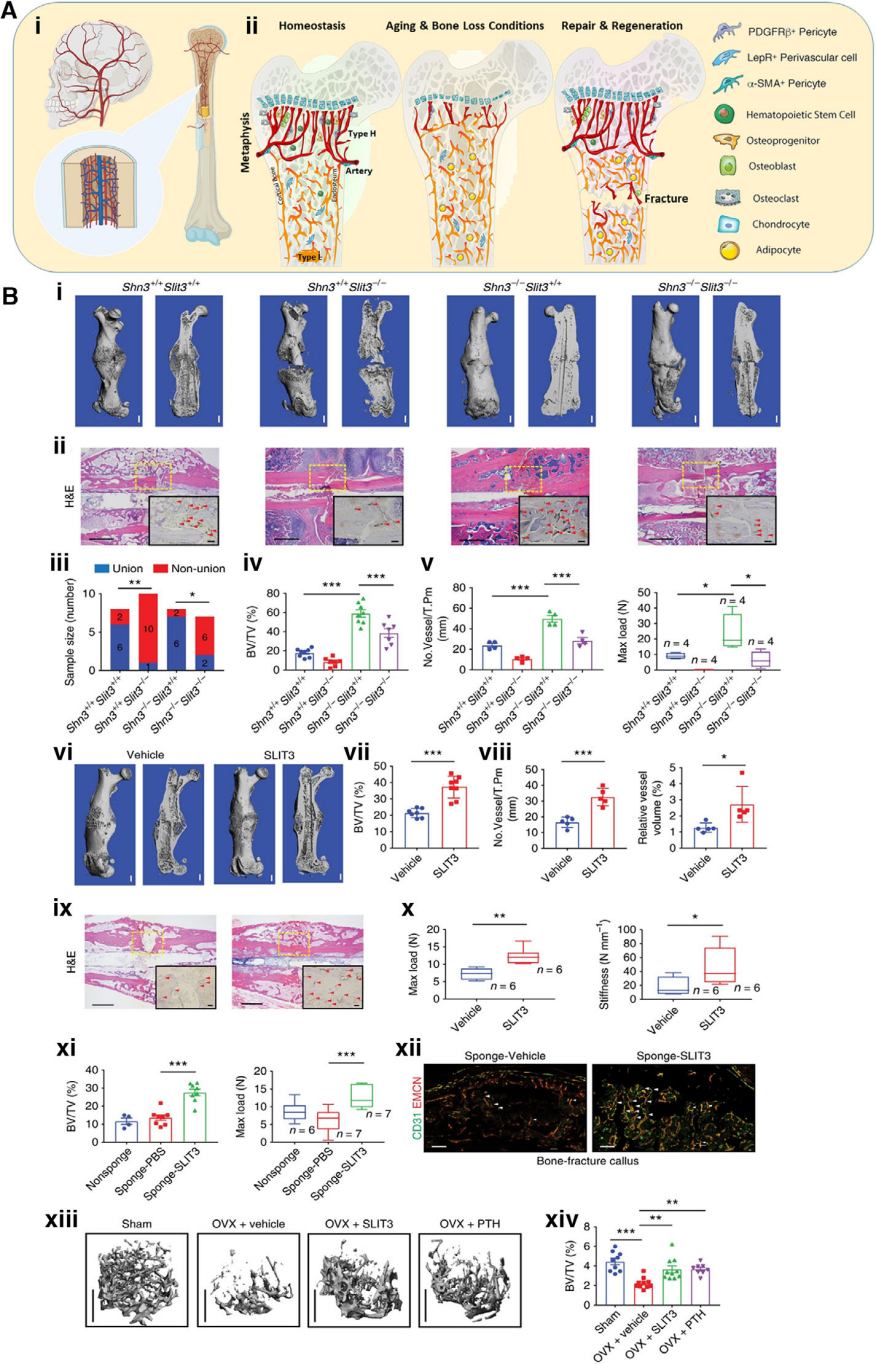
Targeting skeletal endothelium to ameliorate bone loss. (A) (i) The vasculature is widely distributed in the skeleton. (ii) Type H vessels are the primary factor regulating and maintaining perivascular osteoprogenitors and promoting angiogenesis. They are rich in young people (left) and traumatic sites in bone (right), but much less frequent among the elderly (middle). (ii) Reprinted with permission.^[^ ^67^ ^]^ (B) Administration of recombinant SLIT3 has a salutary influence on bone fracture healing, as well as OVX‐induced bone loss in mouse femur. (i) Representative micro‐CT (ii) Representative H&E staining and endomucin (EMCN) immunohistochemistry (IHC) images 3 weeks after open fracture in the midshaft. The boxes represent the fracture site. Arrowheads highlight EMCN‐positive vessels. (iii–v) Non‐union frequency, micro‐CT analysis of BV/TV in callus region. (iv) Shn3^+/+^ Slit3^+/+^, Shn3^+/+^ Slit3^−/−^, Shn3^−/−^ Slit3^+/+^, and Shn3^−/−^ Slit3^−/−^, EMCN‐positive vessel numbers (v, left) and maximum compressive loading (v, right) of the fractured femora 28 days after open midshaft fracture in femur. (vi,vii) Representative micro‐CT (vi) H&E staining (vii) and EMCN IHC images of mouse femurs 3 weeks after fracturing with i.v. injection of SLIT3 or PBS. (viii–x) Micro‐CT measurement of BV/TV in callus area, EMCN‐positive vessel number and volume (ix), and maximum compressive load and stiffness (x) of femurs 3 weeks post‐fracture with i.v. injection of SLIT3 or PBS. (xi) Test of fracture callus BV/TV (left) and maximum loading (right) of mouse femurs excised 3 weeks after fracturing with insertion of a gelatin sponge soaked with SLIT3 or vehicle. (xii) Representative confocal images of CD31 (green) and EMCN (red) dual‐immunostained callus sections of mouse femurs 3 weeks after fracturing with insertion of a gelatin sponge soaked with SLIT3 or vehicle (high power, insert). Arrowheads highlight CD31^hi^EMCN^hi^ vessels. (xiii,xiv) Representative μCT images of the trabecular bone in the distal femur (xiii) and relative BV/TV. Values represent mean ± s.e.m. Reproduced with permission.^[^ ^68^ ^]^ Copyright 2018, Springer Nature

Though the process of bone formation is regulated mainly by OBs, other factors in the skeletal system, such as the vascular endothelium, positively contribute to osteogenesis, supporting bone formation. Vascularization is seen as a premise during bone fracture healing; thus, osteogenesis is not an independent issue of the homeostatic remodeling of bone cells for bone formation. The vasculature is widely distributed in hard tissue and marrow (Figure 5A), and the growth of blood vessels in bone is always coupled with processes such as maintaining perivascular osteoprogenitors. Hence there should be a consensus for therapy about coupling angiogenesis to osteogenesis by targeting the skeletal endothelium.^[^ ^66^ ^]^

The organization and niche microenvironment of blood vessels in the bone marrow is in charge of the regulation of bone homeostasis, aging, and regeneration. Type H vessels are the primary element regulating and maintaining perivascular osteoprogenitors and promoting angiogenesis. They are abundant in the metaphyseal region in young people, but much less frequent in the elder population due to decreased osteogenesis mediated by the reduction of osteoprogenitors (Figure 5A).^[^ ^67^ ^]^ The traumatic bone injury such as a fracture can stimulate the proliferation of type H vessels, osteoprogenitors, and hematopoietic stem cells (HSCs), and accelerate differentiation of progenitor cells, leading to angiogenesis and osteogenesis during bone modeling and remodeling.

Examples of successful bone repair therapies targeting the skeletal endothelium include those reported by Ren Xu,^[^ ^68^ ^]^ in which bone regeneration is promoted by exogenous slit guidance ligand 3(SLIT3), a novel angiogenic factor. A subpopulation of CD31^hi^endomucin^hi^(EMCN^hi^) vascular endothelium was identified as residing in the bone marrow near the growth plate. Ren further identified the role of SLIT3 in bone metabolism, as an osteoblast‐derived regulator of CD31^hi^EMCN^hi^ endothelium. The SLIT3‐dependent crosstalk between osteoblasts and CD31^hi^EMCN^hi^ endothelium improved bone mass and bone fracture healing (Figure 5B). Based on the limitations of current anabolic agents (such as PTH), the development of more novel targets in nanomedicine for bone loss is highly desirable.

### 4.3 Bone targeting and minimally invasive therapeutics

Advanced bone‐targeted diagnosis and treatment of bone loss are vital for the improvement of efficacy and could also reduce costs and recovery time. A bone‐targeting peptide was generated by Jingo^[^ ^69^ ^]^ for biomedical imaging of bone, demonstrating strong specific binding to hydroxyapatite (HA), a major inorganic constituent in skeleton and tooth. This approach could help facilitate the diagnosis and treatment of skeletal diseases such as osteoporosis. There is also new research reporting bone‐targeted delivery of adenosine to combat bone loss from osteoporosis.^[^ ^70^ ^]^ Those researchers generated a bone‐targeted nanocarrier system, containing hyaluronic acid (HA) copolymerized with phenylboronic acid and administration of adenosine, that attenuated ovariectomy (OVX)‐induced bone loss. Bone targeting is supposed to be a kernel for any treatment of bone loss.

Finally, the concept of minimal invasiveness ought to be adhered to in the broader applications of feasible and translational therapy in the clinic. Cells, biomaterials, and biomolecules, applied individually or in combination, could contribute to tissue regeneration in a minimally invasive (nearly non‐invasive) way, but with robust results in terms of regained function and lower risk, fewer complications, and reduced cost. Although some clinical procedures have been involved in some minimally invasive therapeutics, the development of injectable biomaterials should become a priority in the development of regenerative therapies for bone defects, coupled with a deep understanding of bone regeneration biology.

## CONFLICT OF INTEREST

Wei Tao and Omid C. Farokhzad are members of the *Exploration* editorial board. Omid C. Farokhzad has financial interests in Selecta Biosciences, Tarveda Therapeutics, XLINK Therapeutics, PrognomIQ, and Seer.
